# 6-Gingerol Attenuates Ischemia-Reperfusion-Induced Cell Apoptosis in Human AC16 Cardiomyocytes through HMGB2-JNK1/2-NF-*κ*B Pathway

**DOI:** 10.1155/2019/8798653

**Published:** 2019-02-11

**Authors:** Weiyue Zhang, Xiaoyan Liu, Yiping Jiang, Nani Wang, Feng Li, Hailiang Xin

**Affiliations:** ^1^School of Preclinical Medicine, Beijing University of Chinese Medicine, Beijing 100029, China; ^2^Department of Pharmacognosy, School of Pharmacy, Second Military Medical University, Shanghai 200433, China; ^3^Department of Medicine, Zhejiang Academy of Traditional Chinese Medicine, Hangzhou, Zhejiang 310007, China

## Abstract

Myocardial ischemia/reperfusion (I/R) injury is a key factor in deterioration of myocardial function. The c-Jun NH2-terminal kinase (JNK) activation and the transcription factor nuclear factor-kappaB (NF-*κ*B) nuclear translocation have been found in I/R injury. 6-Gingerol, an important bioactive ingredient of ginger, has been reported to have cardiovascular pharmacological effects. However, the molecular mechanism through which it is beneficial is unclear. In this work, I/R induced the increase in the apoptosis and reactive oxygen species level in AC16 cardiomyocytes. 6-Gingerol administration decreased cardiomyocyte apoptosis and improved oxidative stress indexes. 6-Gingerol administration also inhibited I/R-induced HMGB2 expression upregulation and JNK activation and reduced Cleaved Poly(ADP-ribose) polymerases (PARP) and Caspase-3 expression. HMGB2 treatment mimicked the effect of I/R-induced cell damage, which was reversed by 6-gingerol administration. On the other hand, transcriptional activity of NF-*κ*B was reduced in 6-gingerol treated cells. Thus, overall results indicated that 6-gingerol administration protected I/R-induced cardiomyocytes apoptosis via JNK/NF-*κ*B pathway in the regulation of HMGB2. This work supported the efficacy of 6-gingerol on cardiovascular disease and partially revealed its mechanism, which was helpful for understanding the therapeutic effects of this natural drug.

## 1. Introduction

Myocardial infarction is a serious manifestation of cardiovascular disease [1]. Tissue injury and cell death depend on the length of the ischemic period and the reperfusion conditions [2]. Short ischemic periods elicit cardioprotection and longer periods result in irreversible injury. Reperfusion is also harmful, which increases cell mortality by mediating reactive oxygen species (ROS) [3]. It has been known that ischemia/reperfusion (I/R) treatment increased caspase activity and cell apoptosis in cardiomyocytes [4]. Thus, reducing apoptosis has been clinically significant for alleviating myocardial I/R injury.

6-Gingerol is the main bioactive component of ginger (*Zingiber officinal* Roscoe, Zingiberaceae) [5]. It exhibits various pharmacological effects, such as anti-inflammatory and antimutagenic activities [6]. Recently, studies showed that 6-gingerol had significant antioxidative effect in the treatment of cardiovascular diseases [7]. For example, 6-gingerol could prevent atherosclerosis by inhibiting oxidative stress-induced apoptosis. It protected cardiomyocytes apoptosis through antioxidation against doxorubicin-induced injury [8]. 6-Gingerol also inhibited apoptosis in response to I/R injury in the heart [9]. However, mechanisms of 6-gingerol alleviating or eliminating I/R injury in cardiomyocytes remain unexplored.

High-mobility group box (HMGB) family, including HMGB1, HMGB2, and HMGB3, act as proinflammatory mediators [10]. HMGB1 expression increases during infection or injury by activated immune cells and damaged cells. I/R can activate HMGB1 release [11]. The increased endogenous HMGB1 further induces ROS, which can directly stimulate cytotoxic responses and activate NF-*κ*B pathway. HMGB1 also aggravates myocardial I/R injury via JNK1/2 and NF-*κ*B signaling [12]. Study has shown that both HMGB1 and HMGB2 levels increase in the serum of myocardial infarction patients, ischemic myocardial tissues, and hypoxic H9C2 cells. Besides HMGB1, HMGB2 is associated with myocardial infarction severity and induces cell apoptosis in myocardial ischemic animals [13]. HMGB2 overexpression increases cell apoptosis through activating JNK1/2-NF-*κ*B signaling in cardiomyocytes. Based on the above evidence, this study tested the hypothesis that HMGB-JNK1/2-NF-*κ*B signaling might be related to the protective effect of 6-gingerol against I/R injury.

## 2. Material and Method

### 2.1. Cell Culture and Modeling

AC16 cardiomyocytes were cultured in DMEM with 10% fetal bovine serum (Gibco, USA), 1% Penicillin-Streptomycin Solution (100X; Solarbio), and 10 mM glucose and incubated in an incubator containing 5% CO_2_ and 1% O_2_ at 37°C for 24 h. Then, cells were transferred into DMEM containing 10 mM glucose and 10% fetal bovine serum and incubated for 12 h for simulated I/R model.

### 2.2. CCK-8 Assay

AC16 cardiomyocytes were seeded in the 96-well plate (3 × 10^4^ cell/well) and maintained in 5% CO_2_ incubator at 37°C overnight. Then, the wells were divided into several groups: (1) AC16 cardiomyocytes cultured in normal medium (control); (2) AC16 cardiomyocytes following I/R (I/R); and (3) I/R model cells cultured for 24 h in different concentrations of 6-gingerol for 24 h. Cell viability was determined using a CCK-8 assay kit (Beyotime, China) according to the instruction of manufacturer.

### 2.3. Cell Apoptosis Assay

The cells were harvested and stained with annexin V-FITC (Beyotime, China) according to the manufacturer's instructions. After incubation for 20 min at 4°C, the cells were analyzed using flow cytometry.

### 2.4. Western Blotting Assay

Total protein from the cells was extracted using a total protein extraction buffer (Beyotime, China). 10% sodium dodecyl sulfate polyacrylamide gel was used to isolate the proteins. After being transferred to nitrocellulose membrane, the band was blocked with 5% nonfat milk. The blots were incubated with primary antibodies and second antibodies, respectively. The antibodies and reagents were used as follows: HMGB2 (Abcam, Ab124670); JNK1/2 (CST, #9252); P-JNK1/2 (CST, #4668); Cleaved caspase3 (CST, #9661); Cleaved PARP (CST, #9545); NF-*κ*B P65 (Abcam, Ab16502); GAPDH (CST, #5174); H3 (CST, #4499); HRP-labeled Goat Anti-Rabbit IgG (Beyotime, A0208). The results were used to visualize proteins by the enhanced chemiluminescence reagents (Tanon, China).

### 2.5. Quantitative Real-Time PCR

RNA was extracted from AC16 cardiomyocytes using RNeasy Mini Kit (Qiagen, Hilden, Germany). The real-time PCR assay for HMGB2 was as follows: HMGB2 forward, 5′TGACAAAGCTCGCTATGACAGG 3′; HMGB2 reverse, 5′GGAAGAAGGCAGATGGTGGC 3′; GAPDH reverse, 5′AGGCTGTTGTCATACTTC 3′.

### 2.6. ROS Determination

AC16 cardiomyocytes were seeded in the 6-well plate (3 × 10^4^ cell/well) and maintained in 5% CO_2_ incubator at 37°C for 24. Then, the wells were divided into several groups: (1) AC16 cardiomyocytes cultured in normal medium (control); (2) AC16 cardiomyocytes following I/R (I/R); (3) I/R model cells cultured for 24 h in 20*μ*M of 6-gingerol (6-gingerol); (4) I/R model cells cultured for 24 h in 20*μ*M of 6-gingerol and 2.5 ng/mL TNF-*α* (6-gingerol+ TNF-*α*). ROS level of cells wasdetermined using Reactive Oxygen Species Assay Kit according to the manufacturer's instructions (Beyotime, China).

### 2.7. ELISA

The levels of SOD and MDA in AC16 cardiomyocytes were measured by commercial ELISA kit according to the manufacturer's instructions (Jiancheng, China).

### 2.8. Statistical Analysis

All experiments were performed on triplicate samples and in triplicate, and the results were expressed as mean ± SD. Differences between groups were examined for significant differences by ANOVA followed by Tukey's test and values of P < 0.05 or 0.01 were considered to be statistically significant.

## 3. Results

### 3.1. 6-Gingerol Protected AC16 Cardiomyocytes from I/R-Induced Apoptosis

**Figure 1:**
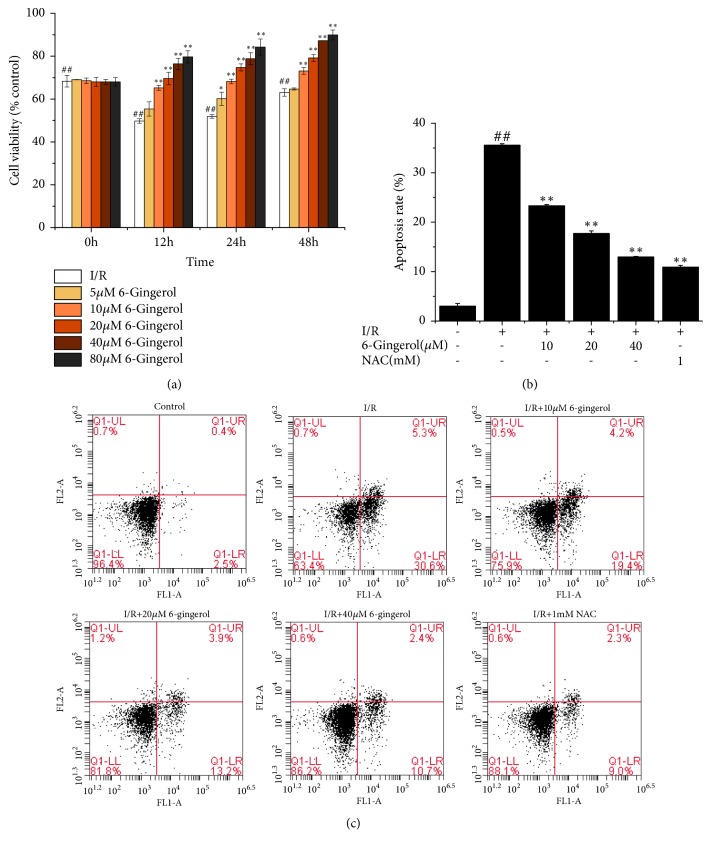
(a) Effect of 6-gingerol on cell viability. (b) Effect of 6-gingerol on cell apoptosis rate. (c) Cell death measured with FCM. ^#^P<0.05, ^##^P<0.01 compared with control group. ^*∗*^P<0.05, ^*∗∗*^P<0.01 compared with I/R group.

The protective effect of 6-gingerol on cardiomyocytes under hypoxia was investigated. As shown in Figure 1, 10-80 *μ*M of 6-gingerol significantly ameliorated hypoxia induced decrease in cell viability. Besides, the results of FCM showed that cell apoptosis induced by hypoxia was markedly revised by 6-gingerol treatment (10-40 *μ*M). It was interesting to find that the effect of 6-gingerol (40 *μ*M) was similar with NAC (1 mM).

### 3.2. 6-Gingerol Reduced ROS Level and Downregulated HMGB2 Expression in AC16 Cardiomyocytes following I/R Injury

**Figure 2:**
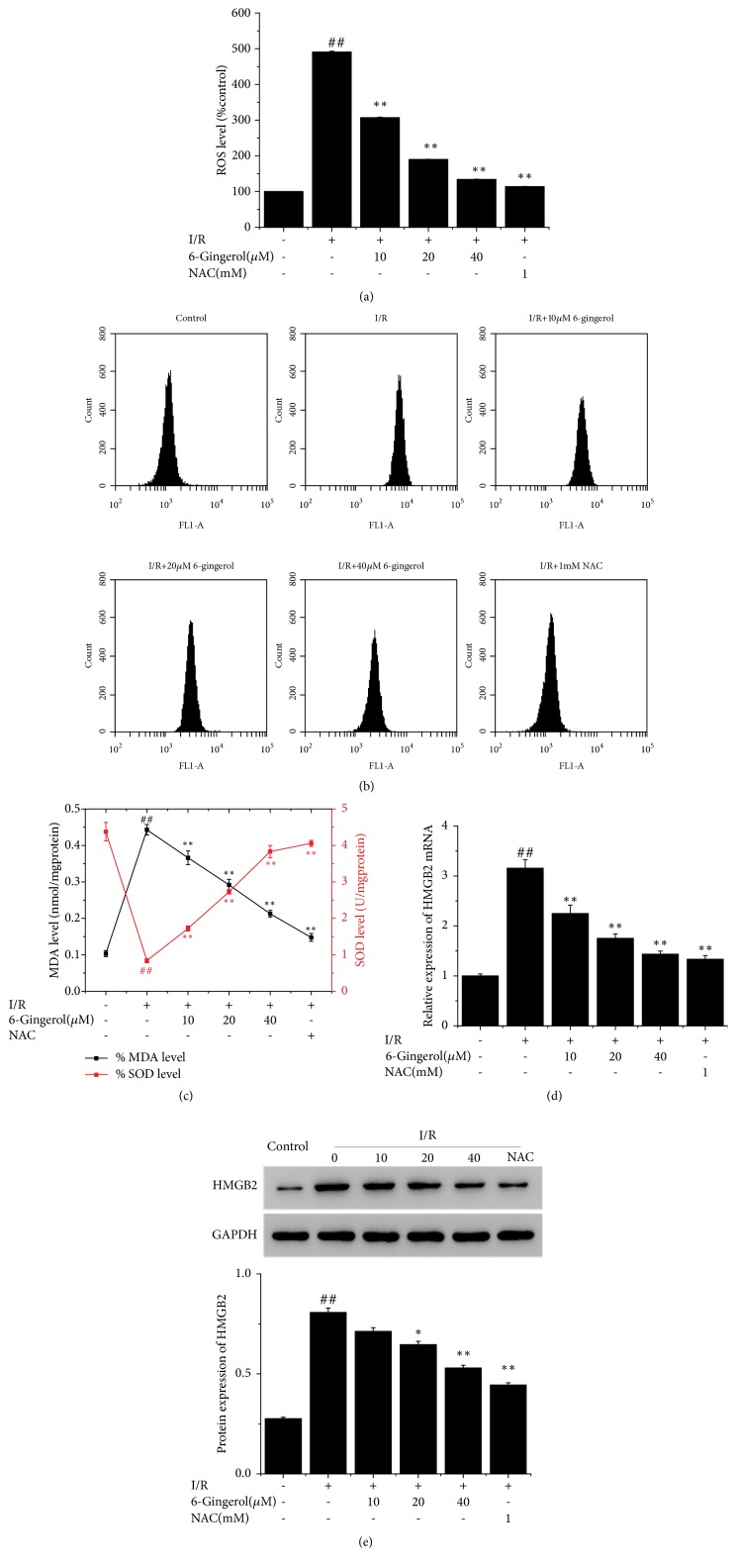
(a) Effect of 6-gingerol on ROS level. (b) ROS measured with FCM. (c) Effect of 6-gingerol on MDA and SOD levels. (d) mRNA expression of HMGB2. (e) Protein expression of HMGB2. ^#^P<0.05, ^##^P<0.01 compared with control group. ^*∗*^P<0.05, ^*∗∗*^P<0.01 compared with I/R group.

To explore the role of ROS in the protective effect of 6-gingerol on AC16 cardiomyocytes following I/R injury, the ROS level in AC16 cells treated with 6-gingerol (10-40*μ*M) was determined by FCM (Figures 2(a) and 2(b)). The ROS level was significantly upregulated by I/R whereas downregulated by the treatment of 6-gingerol. Besides, the MDA level was significantly increased by I/R treatment, whereas the SOD activity was decreased (Figure 2(c)). 6-Gingerol treatment further promoted I/R-induced changes in MDA and SOD.

As shown in Figures 2(d) and 2(e), after being cultured under hypoxia for 24 h, the mRNA levels and protein expression of HMGB2 were significantly upregulated. Treatment with 20-40 *μ*M of 6-gingerol induced a markedly decrease in mRNA levels and protein expression of HMGB2. These results suggested that the protective effect of 6-gingerol on AC16 against hypoxia induced damage might be related to the downregulation of ROS level and HMGB2 expression.

### 3.3. 6-Gingerol Decreased the Activation of JNK 1/2 after the Exposure to HMGB2

**Figure 3:**
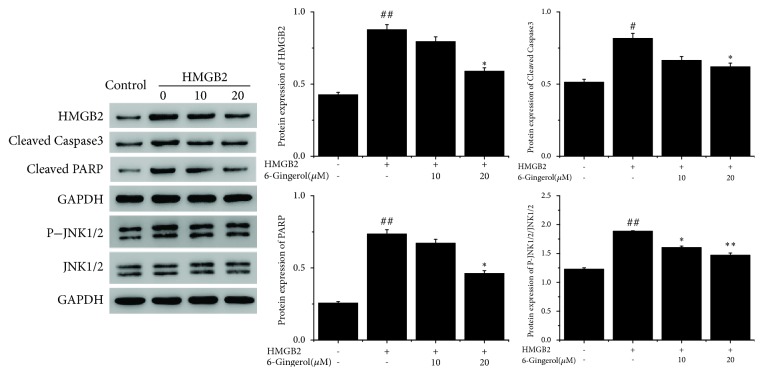
The role of 6-gingerol in protein expression of AC16 cardiomyocytes after exposure to HMGB2. ^#^P<0.05, ^##^P<0.01 compared with control group. ^*∗*^P<0.05, ^*∗∗*^P<0.01 compared with HMGB2 group.

To study the involvement of HMGB2 in the protective effect of 6-gingerol in I/R-induced cell injury, AC16 cardiomyocytes were exposed to HMGB2 (10 ng/mL) with or without treatment of 6-gingerol (10-20*μ*M).  Figure 3 showed that HMGB2 exposure markedly increased the expression of HMGB2, cleaved caspase-3, and PARP, while 6-gingerol treatment significantly inhibited HMGB- induced expression of cleaved caspase-3 and PARP. We also found that AC16 cardiomyocytes with HMGB2 exposure significantly increased the JNK1/2 activation. Then, JNK1/2 activity showed significant alternation after 6-gingerol treatment. These results indicated that 6-gingerol protected AC16 against HMGB2 cytotoxicity via JNK1/2 signaling.

### 3.4. 6-Gingerol Treatment Inhibited NF-*κ*B Nuclear Translocation in I/R-Induced Cells

**Figure 4:**
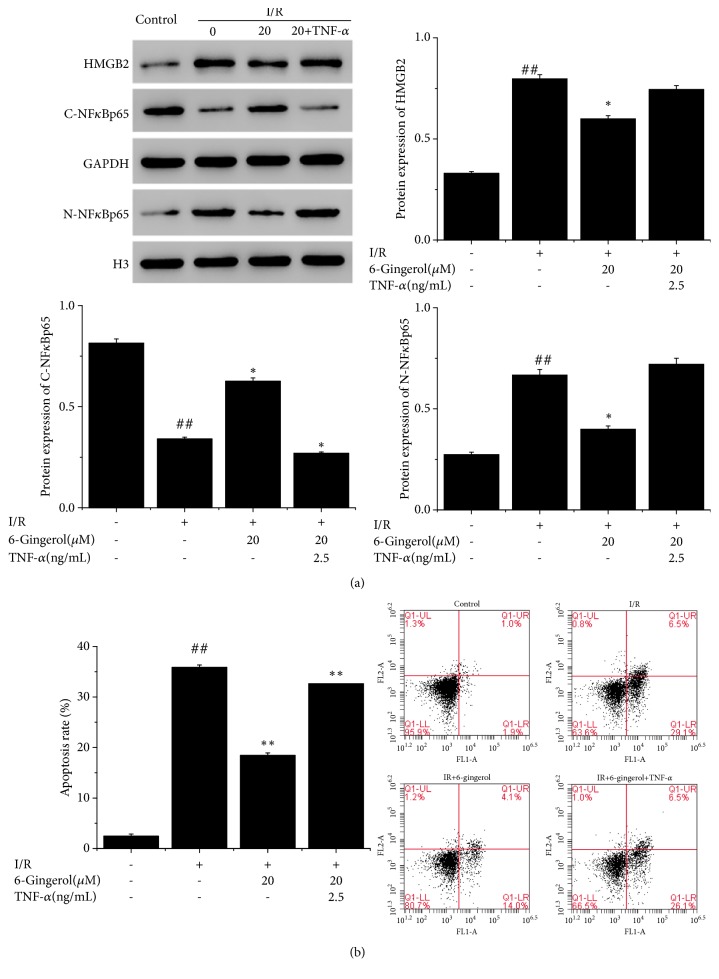
6-Gingerol treatment inhibited I/R-induced (a) NF-*κ*B nuclear translocation and (b) cell apoptosis. ^#^P<0.05, ^##^P<0.01 compared with control group. ^*∗*^P<0.05, ^*∗∗*^P<0.01 compared with I/R group.

NF-*κ*B acted as a putative transcription factor binding the promoter of HMGB2. HMGB2-NF-*κ*B feedback loop regulates cell apoptosis in AC16 cells [14]. To strengthen the results derived from HMGB2 regulation, we examined the effect of 6-gingerol on the NF-*κ*B nuclear translocation in I/R-induced cells. Results demonstrated that compared with the I/R group, 6-gingerol inhibited the NF*κ*B nuclear translocation. To validate it, AC16 cardiomyocytes were treated with TNF-*α* (2.5 ng/mL) as well as 6-gingerol (20*μ*M), and the N-NF*κ*B expression significantly increased. Moreover, TNF-*α* (2.5 ng/mL) treatment increased the apoptosis in AC16 cardiomyocytes (Figure 4). These data suggested that 6-gingerol protected AC16 cardiomyocytes against I/R injury via HMGB2-N-NF-*κ*B feedback loop.

## 4. Discussion

This work reported a novel mechanism of protective effect of 6-gingerol against I/R-induced AC16 cardiomyocytes. The results suggested that 6-gingerol revised cell viability and reduced the apoptosis in I/R-induced cardiomyocytes, reduced ROS and MDA levels, and downregulated HGMB2 expression in I/R-induced injury and protected cardiomyocytes against HGMB2 exposure. It also downregulated the expression of JNK1/2/NF-*κ*B signaling pathway.

6-Gingerol is a phenolic substance extracted from ginger with remarkable antioxidant and antiapoptotic activities. Studies reported that 6-gingerol inhibited ROS production in many cell models, such as lipopolysaccharide stimulated macrophages [15], UVB-induced HaCaT cells [16], human acute T lymphoblastic leukemia MOLT4 cells [17], and so on. Recent researches showed that the combined use of higenamine and 6-gingerol could inhibit doxorubicin-induced oxidative stress and cell apoptosis [18]. It also could inhibit apoptosis to attenuate myocardial I/R injury [9]. In line with the previous study, our results showed the administration of 6-gingerol improved the cell viability and reduced the apoptosis in I/R-induced AC16 cardiomyocytes. I/R treatment was associated with ROS, MDA, and SOD levels, which were dose-dependently recovered by 6-gingerol.

It has been known that increased HMGB2 levels were related to major adverse cardiac events and negatively with ejection fraction in myocardial infarction patients [19]. Similarly, we found that HMGB2 expression increased after I/R in AC16 cardiomyocytes. Although HMGB2 was known primarily as an intracellular protein, activating vascular smooth muscle cells and macrophages promoted the production and release of HMGB2 in response to vascular injury and inflammation. Thus, in addition to the nuclei, HMGB2 was found in the cell supernatants and serum samples of patients [20, 21]. Sequestration of HMGB2 in cells or blocking their release inhibited immune reactions. Additionally, serum HMGB2 levels were closely associated with in-stent restenosis and promoted neointimal hyperplasia with femoral artery injury [20]. In this work, HMGB2 exposure presented similar injury as I/R treatment in cardiomyocytes, which induced cell apoptosis, upregulated Cleaved Caspase3 and PARP, and activated JNK1/2 in AC16 cardiomyocytes. The administration of 6-gingerol not only revised the cell apoptosis but also reduced the expression of HMGB2 and JNK1/2 phosphorylation following HMBG2-induced injury.

JNK1/2/NF-*κ*B signaling pathway was found to be related to many cellular processes in cardiomyocytes, such as cell survival, apoptosis, and angiogenesis [22]. It has been known that the activation of JNK1/2/NF-*κ*B signaling was associated with I/R in AC16 cardiomyocytes [23]. Moreover, HMGB2 silencing inhibited I/R-induced cell apoptosis and JNK1/2 activation. HMGB2 overexpression could be revised by the treatment with NF*κ*B inhibitor [24]. Similar to our finding that both HMGB2 expression and NF-*κ*B nuclear translocation were enhanced by I/R treatment, administration of 6-gingerol downregulated HMGB2 expression and inhibited NF-*κ*B nuclear translocation. Treatment of AC16 cardiomyocytes with TNF-*α* activated NF-*κ*B and this resulted in enhanced expression of HMGB2 and N-NF-*κ*B. It indicated that 6-gingerol reduced NF-*κ*B activation by JNK1/2 signaling with regulation of HMGB2.

## 5. Conclusion

In summary, our study is the first attempt to investigate the possible role of HMGB2-JNK1/2-NF-*κ*B pathway in 6-gingerol protective effect on cardiomyocytes against I/R-induced injury. 6-Gingerol has potential to be a safe and useful option for patients in conditions of cardiovascular risk. Further clinical investigations should be carried out before a conclusion can be reached on the utility of 6-gingerol in this setting.

## Data Availability

The data used to support the findings of this study are available from the corresponding author upon request.
